# GluN2A or GluN2B subunits of the NMDA receptor contribute to changes in neuronal excitability and impairments in LTP in the hippocampus of aging mice but do not mediate detrimental effects of oligomeric Aβ (1–42)

**DOI:** 10.3389/fnagi.2024.1377085

**Published:** 2024-05-20

**Authors:** Nicolina Südkamp, Olena Shchyglo, Denise Manahan-Vaughan

**Affiliations:** Department of Neurophysiology, Medical Faculty, Ruhr University Bochum, Bochum, Germany

**Keywords:** NMDA, GluN2, CA1, amyloid-beta, amyloidosis, Alzheimer, rodent

## Abstract

Studies in rodent models have revealed that oligomeric beta-amyloid protein [Aβ (1–42)] plays an important role in the pathogenesis of Alzheimer’s disease. Early elevations in hippocampal neuronal excitability caused by Aβ (1–42) have been proposed to be mediated via enhanced activation of GluN2B-containing N-methyl-D-aspartate receptors (NMDAR). To what extent GluN2A or GluN2B-containing NMDAR contribute to Aβ (1–42)-mediated impairments of hippocampal function in advanced rodent age is unclear. Here, we assessed hippocampal long-term potentiation (LTP) and neuronal responses 4–5 weeks after bilateral intracerebral inoculation of 8–15 month old GluN2A^+/−^ or GluN2B^+/−^ transgenic mice with oligomeric Aβ (1–42), or control peptide. Whole-cell patch-clamp recordings in CA1 pyramidal neurons revealed a more positive resting membrane potential and increased total spike time in GluN2A^+/−^, but not GluN2B^+/−^-hippocampi following treatment with Aβ (1–42) compared to controls. Action potential 20%-width was increased, and the descending slope was reduced, in Aβ–treated GluN2A^+/−^, but not GluN2B^+/−^ hippocampi. Sag ratio was increased in Aβ–treated GluN2B^+/−^-mice. Firing frequency was unchanged in wt, GluN2A^+/−^, and GluN2B^+/−^hippocampi after Aβ–treatment. Effects were not significantly different from responses detected under the same conditions in wt littermates, however. LTP that lasted for over 2 h in wt hippocampal slices was significantly reduced in GluN2A^+/−^ and was impaired for 15 min in GluN2B^+/−^-hippocampi compared to wt littermates. Furthermore, LTP (>2 h) was significantly impaired in Aβ–treated hippocampi of wt littermates compared to wt treated with control peptide. LTP induced in Aβ–treated GluN2A^+/−^ and GluN2B^+/−^-hippocampi was equivalent to LTP in control peptide-treated transgenic and Aβ–treated wt animals. Taken together, our data indicate that knockdown of GluN2A subunits subtly alters membrane properties of hippocampal neurons and reduces the magnitude of LTP. GluN2B knockdown reduces the early phase of LTP but leaves later phases intact. Aβ (1–42)-treatment slightly exacerbates changes in action potential properties in GluN2A^+/−^-mice. However, the vulnerability of the aging hippocampus to Aβ–mediated impairments of LTP is not mediated by GluN2A or GluN2B-containing NMDAR.

## 1 Introduction

Early changes in the brain during Alzheimer’s disease (AD) arise in part due to the pathophysiological effects of oligomeric Aβ (1–42) (Mucke et al., 2000; Fukumoto et al., 2010; Edwards, 2019). A characteristic feature of oligomeric Aβ (1–42) is the impairment of hippocampal long-term potentiation (LTP), whereby acute effects occur (Wang et al., 2004; Klyubin et al., 2005; Kalweit et al., 2015). Furthermore, deficits in both LTP and learning days after intracerebral treatment with Aβ (1–42) have been reported (Zhang et al., 2017; Khodadadi et al., 2018). Examination of the effects of oligomeric Aβ have indicated that topical application of Aβ to the slice chamber causes a suppression of GABA_A_ receptor function in the hippocampus of young (P25-P40) rats *in vitro* (Orr et al., 2014). It has been proposed that this can lead to elevated levels of extrasynaptic glutamate that, in turn, enable enhanced activation of GluN2B-containing N-methyl-D-aspartate receptors (NMDAR), which then mediate hyperexcitability (Lei et al., 2016). Others have reported that antagonism of GluN2B-containing NMDAR prevents Aβ (1–42)-mediated deficits in LTP in the hippocampal CA1 region of young adult rats (Hu et al., 2009).

LTP in the CA1 region is predominantly NMDAR-dependent and postsynaptically mediated (Malenka et al., 1988, but see also Falcón-Moya et al., 2020 and Grover and Teyler, 1990 for examples of exceptions). NMDAR are typically composed of two GluN1 subunits and two GluN2 subunits (Dingledine et al., 1999). GluN2A and GluN2B-containing NMDAR play a key role in the enablement of hippocampal LTP (Bartlett et al., 2007; Berberich et al., 2007; Ballesteros et al., 2016) Although GluN2C and GluN2D subunits also occur in NMDAR, these do not appear to play a critical role in LTP (Banerjee et al., 2009). Whereas co-agonist binding of glycine or D-serine occurs at the GluN1 subunit (Hirai et al., 1996; Mothet et al., 2001; Henneberger et al., 2010), glutamate binds to the GluN2 subunit (McBain and Mayer, 1994; Laube et al., 1997). GluN2A-containing NMDAR exhibit faster kinetics compared to GluN2B-containing NMDAR (Punnakkal et al., 2012), lose their Mg^2+^ block at lower membrane potentials compared to GluN2B-containing NMDAR (Clarke and Johnson, 2006; Clarke et al., 2013), but allow half as much charge transfer, deactivate faster, and enable less Ca^2+^-influx per unit of current than GluN2B-containing NMDAR (Vicini et al., 1998; Erreger et al., 2005; Sobczyk et al., 2005; Clarke et al., 2013). Furthermore, GluN2A-containing NMDAR respond to weaker stimuli (Köhr et al., 2003; Berberich et al., 2005, 2007) and enable weaker and less persistent forms of LTP compared to GluN2B-containing NMDAR (Ballesteros et al., 2016).

Excessive activation of NMDAR leads to excitotoxity (Rothman and Olney, 1987) and NMDAR antagonists have proven effective in the treatment of cognitive deficits in early AD (Paoletti et al., 2013; Zhou and Sheng, 2013). It is widely believed that the excitotoxic effects of NMDAR in AD are mediated by excessive extracellular glutamate that leads to overactivation of GluN2B-containing NMDAR (Texidó et al., 2011; Danysz and Parsons, 2012; Paoletti et al., 2013; Talantova et al., 2013; Zhou and Sheng, 2013). In addition, NMDAR have been reported to mediate specific cellular and biochemical actions of Aβ in processes that involve both GluN2A and GluN2B subunits (Roselli et al., 2005; Snyder et al., 2005; Domingues et al., 2007; Abbott et al., 2008; Deshpande et al., 2009; Li et al., 2009) in a process that may involve Aβ–binding to NMDAR (Cowburn et al., 1997; De Felice et al., 2007; Lacor et al., 2007).

The contribution of different GluN subunits to NMDAR toxicity, or Aβ–mediated pathophysiology, may change along the lifespan of an individual. Developmental changes in the expression of GluN2A and GluN2B have been reported, whereby a systematic increase of GluN2A subunits and a decline of GluN2B subunits occurs in the period encompassing early postnatal stages (12 days postnatally) through early adulthood (35 days postnatally) (Carmignoto and Vicini, 1992). More recent findings suggest that GluN2A and GluN2B levels remain abundant and largely equivalent in later adulthood (2–4 months postnatally), at least in C57BL/6 mice, although relative differences in murine strains occur (Beckmann et al., 2020). Furthermore, differences in GluN2A:GluN2B ratios occur along the dorsoventral axis of the hippocampus (Dubovyk and Manahan-Vaughan, 2018). GluN2A:GluN2B ratios are also modulated by synaptic activity, whereby lower levels lead to an increase in GluN2B and a decrease in GluN2A subunits (Chen and Bear, 2007; Yashiro and Philpot, 2008). The consequence is a prolongation of NMDAR currents and a reduction in LTP thresholds (Chen and Bear, 2007; Yashiro and Philpot, 2008). Thus, reductions in synaptic activity triggered by Aβ (Balleza-Tapia et al., 2010) may lead to a preferential recruitment of GluN2B-containing NMDAR into synaptic plasticity processes.

In the present study, we explored to what extent GluN2A and GluN2B-containing NMDAR contribute to changes in hippocampal excitability and LTP triggered by intracerebral inoculation with oligomeric Aβ (1–42) in aging mice. We treated 8–15 month old GluN2A^+/−^ and GluN2B^+/−^ animals, and their wt littermates, with oligomeric Aβ (1–42), or control peptide, 4–5 weeks before assessing neuronal excitability and LTP in the hippocampal slice preparation. Effects of Aβ (1–42) on neuronal excitability were minimal. LTP was reduced in GluN2A^+/−^ and GluN2B^+/−^ mice compared to their wt littermates. In wt hippocampi, intracerebral pretreatment with Aβ (1–42) potently reduced the magnitude of LTP. Strikingly, however, pretreatment with Aβ (1–42) had no impact on the profile of LTP expressed in the hippocampi of GluN2A^+/−^ and GluN2B^+/−^ mice. These findings suggest that in old age, the detrimental effects of Aβ (1–42) on LTP are not mediated by GluN2A and GluN2B containing NMDAR.

## 2 Materials and methods

### 2.1 Animals

Eight-to-fifteen month old heterozygote GluN2A (Sakimura et al., 1995) and GluN2B heterozygote (von Engelhardt et al., 2008) transgenic mice and their wildtype littermates (Zentrale Versuchstierhaltung Medizin, Ruhr University Bochum) were used in this study. Homozygotes of GluN2B knockout mice do not survive postnatally (von Engelhardt et al., 2008).

Mice were housed in a custom-made ventilated and acclimatized vivarium in a rodent-housing room (12-h light/dark cycle) with unlimited access to food and water. Experiments were carried out in accordance with the European Communities Council Directive of September 22nd, 2010 (2010/63/EU) for care of laboratory animals, and were conducted according to the guidelines of the German Animal Protection Law. Experiments were authorized in advance by the North Rhine-Westphalia (NRW) State Authority (Landesamt für Arbeitsschutz, Naturschutz, Umweltschutz und Verbraucherschutz, NRW).

### 2.2 Treatment with Aβ (1–42)

Oligomeric Aβ (1–42) was prepared and aggregated as described previously (Kalweit et al., 2015). The soluble Aβ (1–42) peptide was prepared in phosphate-buffered saline at pH 7.4, diluted to a dose of 50 μM, shock-frozen with liquid nitrogen and stored at −80°C. On the day of treatment, the peptide solution was incubated for 3 h to allow for oligomerization (Kalweit et al., 2015). It was applied at room temperature in a dose of 10 μM (1 μL volume) to both lateral cerebral ventricles of anesthetized mice by means of a Hamilton syringe (Kalweit et al., 2015). Control animals received 10 μM scrambled Aβ-peptide (Yamin et al., 2016) in a volume of 1 μL in a procedure that followed identical steps as described above. Treatment was implemented 4–5 weeks prior to conducting the *in vitro* experiments.

### 2.3 Slice preparation

Mice were deeply anaesthetized with isoflurane before decapitation and sagittal hippocampal slices (350 μm) were prepared in cold (1–4°C), oxygenated saccharose solution (in mM: 87 NaCl, 2.6 MgSO₄, 75 Saccharose, 2.5 KCl, 1.25 NaH₂PO₄, 26 NaHCO₃, 0.5 CaCl₂, 2 D-Glucose) (95% O₂, 5% CO₂). Slices were subsequently incubated, for at least 30 min before recordings were commenced, in a holding chamber in artificial cerebrospinal fluid (aCSF, in mM: 125 mM NaCl, 3 mM KCl, 2.5 mM CaCl₂, 1.3 mM MgSO₄, 1.25 mM NaH₂PO₄, 26 mM NaHCO₃ and 13 mM D-Glucose) using a constant flow rate of 2 mL/min at 30°C.

### 2.4 Patch clamp recordings

Whole cell patch clamp recordings were conducted according to established procedures (Novkovic et al., 2015). The recording chamber was located under an upright microscope. Slices were continuously perfused with oxygenated aCSF (constant flow rate of 1–2 mL/min). Recording pipettes were prepared from borosilicate glass tubes (1.5 mm external diameter) with a resistance of 6–10 MΩ and were filled with intracellular solution (in mM: 97.5 potassium gluconate, 32.5 KCl, 5 EGTA, 10 Hepes, 1 MgCl_2_, 4 Na_2_ ATP, adjusted to pH 7.3 with KOH). Patch clamp recordings were conducted on visually identified soma of pyramidal neurons in the CA1 region. Corrections related to the liquid junction potential (Neher, 1992) were not conducted.

**Figure 1:**
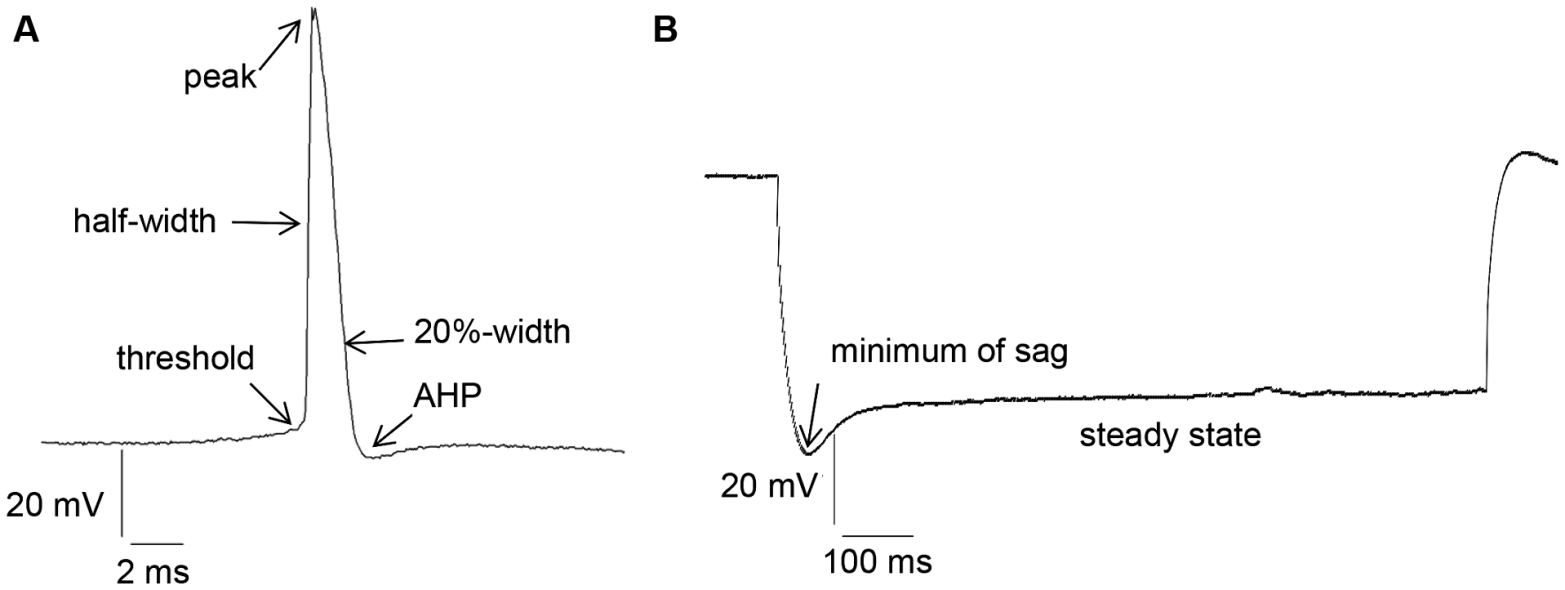
**(A)** Examples of action potential (AP) measurement: threshold, half-width, peak, 20%-width and afterhyperpolarization (AHP). **(B)** Measurement of Sag and Sag ratio: Sag ratio was determined from the ratio between the steady-state decrease in voltage and the greatest decrease in voltage after a hyperpolarizing current step.

Intrinsic membrane properties were assessed using an HEKA EPC10 amplifier and the PATCHMASTER acquisition software (HEKA Elektronik Dr. Schulze GmbH, Lambrecht/Pfalz, Germany). We scrutinized resting membrane potential, input resistance, membrane time constant, excitatory threshold, Sag, sag ratio, firing frequency, action potential (AP) threshold, spike amplitude, AP peak, half-width, 20%-width, time-to-peak, afterhyperpolarization (AHP), time peak to AHP (Figure 1A). Sag ratio was determined as the ratio between the steady-state decrease in voltage and the greatest decrease in voltage after a hyperpolarizing current step, i.e., steady state voltage/peak voltage (Figure 1B).

Data underwent low-pass filtering at 2.9 kHz and were digitized at 10 kHz. FITMASTER software (HEKA Elektronik Dr. Schulze GmbH, Lambrecht/Pfalz, Germany) was used for offline data analysis. Input resistance was calculated from the slope of the linear fit of the relationship between the change in membrane potential (∆V) and the intensity of the injected current (between −120 pA and + 90 pA). The time constant was determined from an exponential fit of the averaged voltage decay. The resting membrane potential was determined from the mean of 30 s basal recording time. The minimum current needed to induce an action potential was defined as the threshold current. The action potential amplitude was measured as the voltage difference between the threshold and the peak. Firing properties were investigated by applying current steps of ∆50 pA in hyperpolarizing and depolarizing square pulses (1-s duration) through the patch-clamp electrode (in the range of −300 pA to 400 pA). Here, we calculated both the absolute number of spikes during the current application, the firing frequency (in Hz), and the spike frequency adaptation. The latter was determined by counting the number of spikes separately during each 100 ms of the 1 s depolarizing square pulse of 300 pA and converting the number into a frequency in Hz.

### 2.5 fEPSP recordings and induction of LTP

To record field potentials, we placed a bipolar stimulation electrode (Fredrick Haer, Bowdowinham, ME, United States) in the stratum radiatum of the CA1 region of the hippocampus and a glass field recording electrode (impedance: 1–2 MΩ, filled with aCSF) was placed in the CA1 dendritic area.

Field excitatory post-synaptic potentials (fEPSPs) were evoked by means of test-pulse stimuli (0.025 Hz, 0.2 ms duration, sample rate of 10,000 Hz). For each time-point, five fEPSPs were averaged. Before recordings were started, a stimulus–response relationship was determined using a stimulation intensity range of 60–660 μA (50 μA steps). The stimulation strength used for test-pulses was the intensity that evoked *ca.* 50% of the maximal fEPSP. Basal synaptic transmission was recorded for 40 min, after which period LTP was induced by theta burst stimulation (TBS, three trains 10 s apart, each consisting of 10 bursts of 4 pulses at 100 Hz, delivered 100 ms apart; Novkovic et al., 2015).

### 2.6 Statistical analysis

Analysis of variance (ANOVA) with repeated measures, or a Student’s t-test was used for statistical analysis. Where appropriate, a *post-hoc* Fischer’s test was used to determine if statistical significances occurred between two individual test conditions. Data are expressed as the mean ± standard error of the mean. ‘N’ signifies the number of animals and ‘n’ signifies the number of hippocampal slices (LTP experiments), or cells (for patch clamp data).

## 3 Results

### 3.1 Aging wildtype mice exhibited a higher input resistance after Aβ-treatment. Other membrane properties were largely unchanged

**Figure 2:**
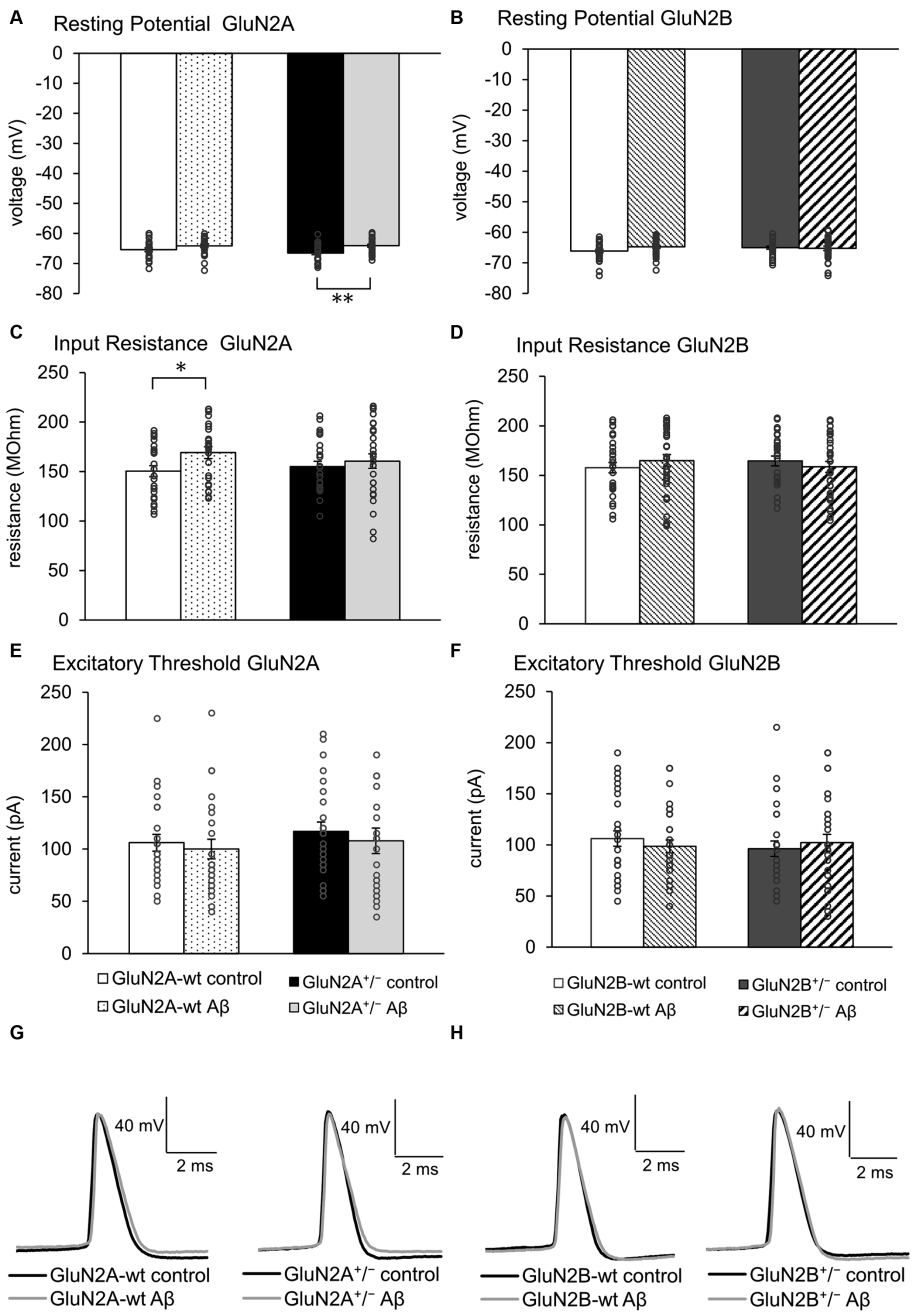
Effects of Aβ-treatment on membrane properties of hippocampal neurons. **(A)** Resting (membrane) potential was not different in GluN2A^+/−^ mice (*N* = 5, *n* = 25) compared to their wt littermates (*N* = 5, *n* = 24) under control conditions. Aβ-treated GluN2A^+/−^ mice (*N* = 5, *n* = 26) exhibited a significant difference in responses compared to control GluN2A^+/−^. This effect derived moreso from the more negative resting potential in control transgenic mice, than a direct effect of oligomeric Aβ (1–42) on the potential. See Tables 1, 2 for statistics. **(B)** Resting (membrane) potential was not different in wt littermates of GluN2B^+/−^ mice following Aβ-treatment (*N* = 6, *n* = 31) compared to control peptide-treated wt (*N* = 6, *n* = 29). GluN2B^+/−^ mice exhibited a similar resting membrane potential following control peptide-treatment (*N* = 5, *n* = 28) compared to control wt. Following Aβ-treatment, no difference in membrane potential was evident when effects in GluN2B^+/−^ transgenics (*N* = 6, *n* = 31) were compared with control peptide-treated GluN2B^+/−^transgenics. See Tables 1, 2 for statistics. **(C,D)** Input resistance was higher in Aβ-treated wt littermates of GluN2A^+/−^ compared to control wt **(C)**. This effect was absent in Aβ-treated GluN2A^+/−^ transgenics compared to control GluN2A^+/−^ mice **(C)**. No effect of Aβ-treatment was detected in GluN2B^+/−^transgenics or their wt littermates **(D)**. See Tables 1, 2 for statistics. **(E,F)** Excitatory threshold was unaffected by Aβ-treatment of GluN2A^+/−^ transgenics **(E)** or GluN2B^+/−^ transgenics **(F)** or their wildtype littermates **(E,F)**. See Tables 1, 2 for statistics. **(G,H)** Representative examples of action potentials in control peptide and Aβ-treated GluN2A^+/−^ transgenics and their wt littermates **(G)** and in control peptide and Aβ-treated GluN2B^+/−^ transgenics and their wt littermates **(H)**. The circles on the error bars show the distribution of individual responses in each condition that contributed the mean effect represented by the bar.

Given that little is known about the response of aging hippocampi to intracerebral treatment with oligomeric Aβ (1–42), we first compared the effect of Aβ-treatment with control peptide-treatment in the wildtype (wt) littermates of GluN2A^+/−^ (control-treated *N* = 5, *n* = 24; Aβ-treated *N* = 5, *n* = 24) and GluN2B^+/−^ mice (control-treated *N* = 6, *n* = 29; Aβ-treated *N* = 6, *n* = 31). Following Aβ-treatment of these two different wt littermate cohorts (Figures 2A,B), no changes in resting potential were evident (Tables 1A, 2A).

**Table 1:** Passive and active neuronal properties in GluN2A^+/−^ mice and their wildtype littermates after Aβ or control peptide treatment.

A						
	GluN2A-wt control	GluN2A-wt Aβ	*T*-test^$^/ANOVA	GluN2A^+/−^ control	GluN2A^+/−^ Aβ	*T*-test^$^/ANOVA
Resting potential (mV)	−65.42 ± 0.59	−64.14 ± 0.58	*p* = 0.14^**$**^	−66.52 ± 0.56	−64.07 ± 0.49	***p* = 0.002**^**$**^
Input resistance (MΩ)	150.37 ± 5.55	169.11 ± 5.94	***p* = 0.029**^**$**^	154.96 ± 5.45	160.59 ± 7.39	*p* = 0.55^**$**^
Tau (ms)	14.73 ± 0.61	15.24 ± 0.73	*p* = 0.604^**$**^	14.01 ± 0.67	14.86 ± 0.61	*p* = 0.36^**$**^
Excitatory threshold (pA)	106.04 ± 8.07	100 ± 9.38	*p* = 0.63^**$**^	117 ± 8.85	107.88 ± 12.18	*p* = 0.56^**$**^
Firing frequency 50pA	0.04 ± 0.04	0.21 ± 0.17	*F* (3,95) = 1.74, *p* = 0.16	0 ± 0	0.12 ± 0.06	*F* (3,95) = 1.74, *p* = 0.16
Firing frequency 100pA	1.04 ± 0.57	1.92 ± 0.57	*F* (3,95) = 1.74, *p* = 0.16	0.44 ± 0.16	1.46 ± 0.47	*F* (3,95) = 1.74, *p* = 0.163
Firing frequency 150pA	3.21 ± 0.90	4.21 ± 0.80	*F* (3,95) = 1.74, *p* = 0.16	1.76 ± 0.32	2.73 ± 0.59	*F* (3,95) = 1.74, *p* = 0.16
Firing frequency 200pA	5.38 ± 1.09	4.88 ± 0.86	*F* (3,95) = 1.74, *p* = 0.16	3.2 ± 0.45	3.73 ± 0.63	*F* (3,95) = 1.74, *p* = 0.16
Firing frequency 250pA	6.13 ± 1.14	5.58 ± 0.82	*F* (3,95) = 1.74, *p* = 0.16	3.76 ± 0.46	4.35 ± 0.70	*F* (3,95) = 1.74, *p* = 0.16
Firing frequency 300pA	6.67 ± 1.21	5.46 ± 0.71	*F* (3,95) = 1.74, *p* = 0.16	4.2 ± 0.37	4.65 ± 0.64	*F* (3,95) = 1.74, *p* = 0.16
Firing frequency 350pA	6.86 ± 1.12	5.29 ± 0.74	*F* (3,95) = 1.74, *p* = 0.16	4.2 ± 0.42	4.92 ± 0.58	*F* (3,95) = 1.74, *p* = 0.16
Firing frequency 400pA	6.84 ± 1.00	5.38 ± 0.69	*F* (3,95) = 1.74, *p* = 0.16	4.52 ± 0.40	4.81 ± 0.49	*F* (3,95) = 1.74, *p* = 0.16
Sag (mV)	−10.29 ± 0.68	−12.55 ± 0.77	***p* = 0.037**^**$**^	−10.96 ± 0.63	−11.46 ± 0.76	*p* = 0.63^**$**^
Sag ratio	0.910 ± 0.005	0.895 ± 0.005	*p* = 0.05^**$**^	0.907 ± 0.005	0.902 ± 0.005	*p* = 0.47^**$**^

B						
	GluN2A-wt control	GluN2A^+/−^ control	*T*-test^$^/ANOVA	GluN2A-wt Aβ	GluN2A^+/−^ Aβ	*T*-test/ANOVA
Resting potential (mV)	−65.42 ± 0.59	−66.52 ± 0.56	p = 0.195^**$**^	−64.14 ± 0.58	−64.07 ± 0.49	*p* = 0.93^**$**^
Input resistance (MΩ)	150.37 ± 5.55	154.96 ± 5.45	*p* = 0.57^**$**^	169.11 ± 5.94	160.59 ± 7.39	p = 0.39^**$**^
Tau (ms)	14.73 ± 0.61	14.01 ± 0.67	*p* = 0.44^**$**^	15.24 ± 0.73	14.86 ± 0.61	*p* = 0.698^**$**^
Excitatory threshold (pA)	106.04 ± 8.07	117 ± 8.85	*p* = 0.38^**$**^	100 ± 9.38	107.88 ± 12.18	*p* = 0.62^**$**^
Firing frequency 50pA	0.04 ± 0.04	0 ± 0	*F* (3,95) = 1.74, *p* = 0.16	0.21 ± 0.17	0.12 ± 0.06	*F* (3,95) = 1.74, *p* = 0.16
Firing frequency 100pA	1.04 ± 0.57	0.44 ± 0.16	*F* (3,95) = 1.74, *p* = 0.16	1.92 ± 0.57	1.46 ± 0.47	*F* (3,95) = 1.74, *p* = 0.16 3
Firing frequency 150pA	3.21 ± 0.90	1.76 ± 0.32	*F* (3,95) = 1.74, *p* = 0.16	4.21 ± 0.80	2.73 ± 0.59	*F* (3,95) = 1.74, *p* = 0.16
Firing frequency 200pA	5.38 ± 1.09	3.2 ± 0.45	*F* (3,95) = 1.74, *p* = 0.16	4.88 ± 0.86	3.73 ± 0.63	*F* (3,95) = 1.74, *p* = 0.16
Firing frequency 250pA	6.13 ± 1.14	3.76 ± 0.46	*F* (3,95) = 1.74, *p* = 0.16	5.58 ± 0.82	4.35 ± 0.70	*F* (3,95) = 1.74, *p* = 0.16
Firing frequency 300pA	6.67 ± 1.21	4.2 ± 0.37	*F* (3,95) = 1.74, *p* = 0.16	5.46 ± 0.71	4.65 ± 0.64	*F* (3,95) = 1.74, *p* = 0.16
Firing frequency 350pA	6.86 ± 1.12	4.2 ± 0.42	*F* (3,95) = 1.74, *p* = 0.16	5.29 ± 0.74	4.92 ± 0.58	*F* (3,95) = 1.74, *p* = 0.16
Firing frequency 400pA	6.84 ± 1.00	4.52 ± 0.40	*F* (3,95) = 1.74, *p* = 0.16	5.38 ± 0.69	4.81 ± 0.49	*F* (3,95) = 1.74, *p* = 0.16
Sag (mV)	−10.29 ± 0.68	−10.96 ± 0.63	*p* = 0.48^**$**^	−12.55 ± 0.77	−11.46 ± 0.76	*p* = 0.33^**$**^
Sag ratio	0.910 ± 0.005	0.907 ± 0.005	*p* = 0.64^**$**^	0.895 ± 0.005	0.902 ± 0.005	*p* = 0.37^**$**^

(A) The table compares passive and active neuronal properties of Aβ (1–42) treatment versus control peptide in either GluN2A^+/−^ mice or their wt littermates. Firing frequencies evoked with currents in the range of 50 through 400pA are shown. Responses obtained in GluN2A^+/−^ mice and their wildtype littermates following treatment with Aβ or control peptide are compared. Significant effects are highlighted in bold. (B) The table compares passive and active neuronal properties in GluN2A^+/−^ mice versus their wt littermates following treatment with either control peptide or Aβ (1–42). Firing frequencies evoked with currents in the range of 50 though 400pA are shown. Responses in GluN2A^+/−^ mice and their wildtype littermates after injections of either Aβ or control injections are compared.

**Table 2:** Passive and active neuronal properties in GluN2B^+/−^ mice and their wildtype littermates following Aβ or control peptide treatment.

A						
	GluN2B-wt control	GluN2B-wt Aβ	*T*-test^$^/ANOVA	GluN2B^+/−^ control	GluN2B^+/−^ Aβ	*T*-test^$^/ANOVA
Resting potential (mV)	−66.14 ± 0.55	−64.75 ± 0.51	*p* = 0.07^**$**^	−65.02 ± 0.50	−65.23 ± 0.73	*p* = 0.82^**$**^
Input resistance (MΩ)	157.72 ± 5.27	164.77 ± 6.14	*p* = 0.398^**$**^	164.50 ± 4.96	158.48 ± 5.52	*p* = 0.43^**$**^
Tau (ms)	12.71 ± 0.56	13.13 ± 0.46	*p* = 0.57^**$**^	13.34 ± 0.75	14.28 ± 0.72	*p* = 0.38^**$**^
Excitatory threshold (pA)	106.21 ± 7.51	98.55 ± 6.26	*p* = 0.44^**$**^	96.25 ± 7.55	102.10 ± 8.24	*p* = 0.61^**$**^
Firing frequency 50pA	0.38 ± 0.21	0.03 ± 0.03	*F* (3,115) = 1.26, *p* = 0.29	0.36 ± 0.25	0.68 ± 0.35	*F* (3,115) = 1.26, *p* = 0.29
Firing frequency 100pA	3.24 ± 0.92	2 ± 0.48	*F* (3,115) = 1.26, *p* = 0.29	3.71 ± 0.97	3.65 ± 1.02	*F* (3,115) = 1.26, *p* = 0.29
Firing frequency 150pA	5.79 ± 1.14	4.23 ± 0.67	*F* (3,115) = 1.26, *p* = 0.29	6 ± 1.24	6.13 ± 1.26	*F* (3,115) = 1.26, *p* = 0.29
Firing frequency 200pA	7.59 ± 1.29	5.58 ± 0.76	*F* (3,115) = 1.26, *p* = 0.29	7.29 ± 1.24	8.06 ± 1.32	*F* (3,115) = 1.26, *p* = 0.29
Firing frequency 250pA	7.83 ± 1.29	6.13 ± 0.75	*F* (3,115) = 1.26, *p* = 0.29	7.68 ± 1.28	9.03 ± 1.32	*F* (3,115) = 1.26, *p* = 0.29
Firing frequency 300pA	8.10 ± 1.28	6.03 ± 0.63	*F* (3,115) = 1.26, p = 0.29	7.39 ± 1.14	9.35 ± 1.30	*F* (3,115) = 1.26, *p* = 0.29
Firing frequency 350pA	7.69 ± 1.25	5.87 ± 0.59	*F* (3,115) = 1.26, *p* = 0.29	7 ± 1.01	9.16 ± 1.19	*F* (3,115) = 1.26, *p* = 0.29
Firing frequency 400pA	7.31 ± 1.16	5.68 ± 0.50	*F* (3,115) = 1.26, *p* = 0.29	7 ± 0.85	8.97 ± 1.09	*F* (3,115) = 1.26, *p* = 0.29
Sag (mV)	−11.06 ± 0.54	−11.02 ± 0.46	*p* = 0.96^**$**^	−12.25 ± 0.64	−10.24 ± 0.77	*p* = 0.056^**$**^
Sag ratio	0.908 ± 0.004	0.907 ± 0.003	*p* = 0.91^**$**^	0.897 ± 0.005	0.913 ± 0.006	***p* = 0.0496**^**$**^

B						
	GluN2B-wt control	GluN2B^+/−^ control	*T*-test^$^/ANOVA	GluN2B-wt Aβ	GluN2B^+/−^ Aβ	*T*-test^$^/ANOVA
Resting potential (mV)	−66.14 ± 0.55	−65.02 ± 0.50	*p* = 0.15^**$**^	−64.75 ± 0.51	−65.23 ± 0.73	*p* = 0.60^**$**^
Input resistance (MΩ)	157.72 ± 5.27	164.50 ± 4.96	*p* = 0.36^**$**^	164.77 ± 6.14	158.48 ± 5.52	*p* = 0.46^**$**^
Tau (ms)	12.71 ± 0.56	13.34 ± 0.75	*p* = 0.51^**$**^	13.13 ± 0.46	14.28 ± 0.72	*p* = 0.19^**$**^
Excitatory threshold (pA)	106.21 ± 7.51	96.25 ± 7.55	*p* = 0.36^**$**^	98.55 ± 6.26	102.10 ± 8.24	*p* = 0.74^**$**^
Firing frequency 50pA	0.38 ± 0.21	0.36 ± 0.25	*F* (3,115) = 1.26, *p* = 0.29	0.03 ± 0.03	0.68 ± 0.35	*F* (3,115) = 1.26, *p* = 0.29
Firing frequency 100pA	3.24 ± 0.92	3.71 ± 0.97	*F* (3,115) = 1.26, *p* = 0.29	2 ± 0.48	3.65 ± 1.02	*F* (3,115) = 1.26, *p* = 0.29
Firing frequency 150pA	5.79 ± 1.14	6 ± 1.24	*F* (3,115) = 1.26, *p* = 0.29	4.23 ± 0.67	6.13 ± 1.26	*F* (3,115) = 1.26, *p* = 0.29
Firing frequency 200pA	7.59 ± 1.29	7.29 ± 1.24	*F* (3,115) = 1.26, *p* = 0.29	5.58 ± 0.76	8.06 ± 1.32	*F* (3,115) = 1.26, *p* = 0.29
Firing frequency 250pA	7.83 ± 1.29	7.68 ± 1.28	*F* (3,115) = 1.26, *p* = 0.29	6.13 ± 0.75	9.03 ± 1.32	*F* (3,115) = 1.26, *p* = 0.29
Firing frequency 300pA	8.10 ± 1.28	7.39 ± 1.14	*F* (3,115) = 1.26, *p* = 0.29	6.03 ± 0.63	9.35 ± 1.30	*F* (3,115) = 1.26, *p* = 0.29
Firing frequency 350pA	7.69 ± 1.25	7 ± 1.01	*F* (3,115) = 1.26, *p* = 0.29	5.87 ± 0.59	9.16 ± 1.19	*F* (3,115) = 1.26, *p* = 0.29
Firing frequency 400pA	7.31 ± 1.16	7 ± 0.85	*F* (3,115) = 1.26, *p* = 0.29	5.68 ± 0.50	8.97 ± 1.09	*F* (3,115) = 1.26, *p* = 0.29
Sag (mV)	−11.06 ± 0.54	−12.25 ± 0.64	*p* = 0.17^**$**^	−11.02 ± 0.46	−10.24 ± 0.77	*p* = 0.39^**$**^
Sag Ratio	0.908 ± 0.004	0.897 ± 0.005	*p* = 0.105^**$**^	0.907 ± 0.003	0.913 ± 0.006	*p* = 0.40^**$**^

(A) The table compares passive and active neuronal properties of Aβ (1–42) treatment versus control peptide in either GluN2B^+/−^ mice or their wt littermates. Firing frequencies evoked with currents in the range of 50 though 400pA are shown. Responses in GluN2B^+/−^ mice and their wildtype littermates following treatment with Aβ or control peptide are compared. Significant effects are highlighted in bold. (B) The table compares passive and active neuronal properties in GluN2B^+/−^ mice versus their wt littermates following treatment with either control peptide or Aβ (1–42). Firing frequencies evoked with currents in the range of 50 though 400pA are shown. Responses in GluNB^+/−^ mice and their wildtype littermates treatment with either Aβ or control peptide are compared.

With the exception of input resistance, which was increased in GluN2A wt littermates (Figure 2C), but unchanged in GluN2B wt littermates after Aβ-treatment (Figure 2D), no other neuronal property was affected by Aβ-treatment in the wt littermates of either transgenic strain (Figures 2D–F; Tables 1A, B, 2A, B).

### 3.2 GluN2 subunit deletion and Aβ-treatment differentially affected the resting membrane potential in GluN2A^+/−^ but not GluN2B^+/−^ mice. Other membrane properties were unaffected by Aβ

Four to five weeks after intracerebral treatment, we observed that control peptide-treated GluN2A^+/−^ mice (*N* = 5, *n* = 25) exhibited an equivalent resting membrane potential (*p* = 0.195) compared to that seen in wt controls (Figure 2A; Table 1B). After treatment with Aβ (1–42) (*N* = 5, *n* = 26), resting membrane potential became more positive (*p* = 0.002) in GluN2A^+/−^ mice, compared to control peptide-treated GluN2A^+/−^ hippocampi (Figure 2A; Table 1A), although the membrane voltage was very similar to responses evoked in Aβ-treated wt hippocampi (Figure 2A). This suggests that the effect of Aβ in GluN2A^+/−^ hippocampi may have derived from the change in membrane potential in the transgenic mice, rather than due to a direct effect of Aβ.

In GluN2B^+/−^ hippocampi, we detected no changes in resting membrane potential following control peptide treatment compared to effects detected in their wt littermates (*p* = 0.15, *N* = 5, *n* = 28) (Figure 2B; Table 2B). Levels achieved were also similar to the resting membrane potential detected in Aβ-treated wt mice (Figure 2B; Table 2B). In addition, treatment with Aβ had no significant effect on resting membrane potential in GluN2B^+/−^ hippocampi (*N* = 6, *n* = 31) compared to control peptide-treated GluN2B^+/−^ hippocampi (Figure 2B; Table 2A).

Input resistance (Figures 2C,D) and excitatory threshold (Figures 2C,D) were unaffected by Aβ-treatment of GluN2A^+/−^ or GluN2B^+/−^ mice compared to control peptide treatment of each transgenic group (Tables 1A, B, 2A, B).

### 3.3 Sag was unaltered after Aβ-treatment of transgenic mice. Sag ratio was increased by Aβ-treatment of GluN2B transgenics, but not of GluN2B wild type littermates

Sag reflects a rebound depolarization that is enabled by hyperpolarization-activated cation currents (I_h_) that are mediated by the opening of hyperpolarization-activated cation non-selective (HCN) channels (Robinson and Siegelbaum, 2003). This process serves to limit the negativity of the resting membrane potential and to regulate synaptic transmission. Given that we detected changes in the resting membrane potential in the abovementioned experiments, we wondered if deletion of a GluN2 subunit or Aβ-treatment affects sag.

**Figure 3:**
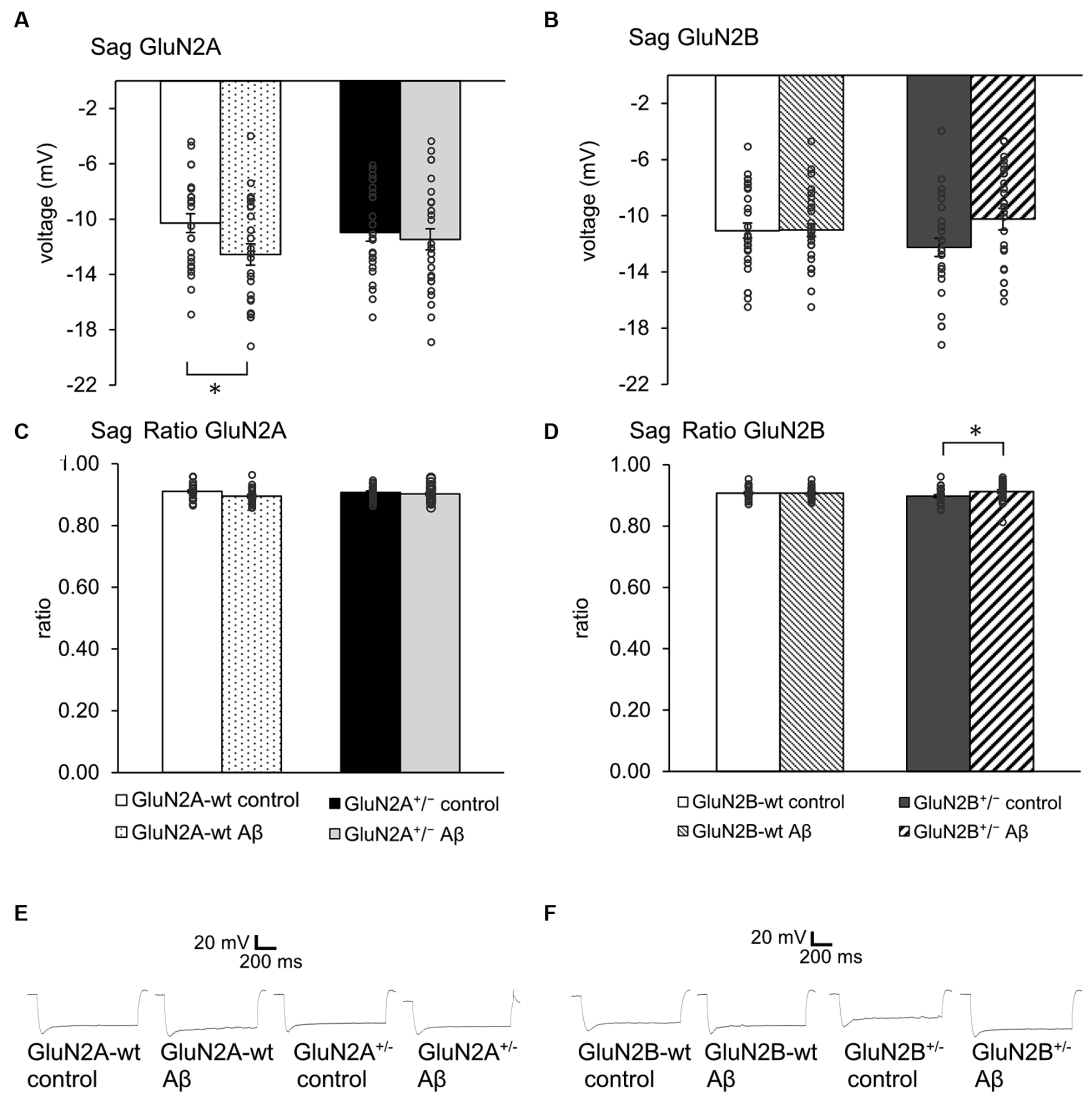
Aβ-treatment selectively altered sag in GluN2A wt littermates and GluN2B^+/−^ **(A,C)**. Sag **(A)**, but not Sag ratio **(C)** was more negative in Aβ-treated wt littermates of GluN2A^+/−^ mice (*N* = 5, *n* = 24) compared to control wt (*N* = 5, *n* = 24), but was unaffected in control peptide -treated GluN2A^+/−^ (*N* = 5, *n* = 25) compared to oligomeric Aβ (1–42)-treated GluN2A^+/−^ (*N* = 5, *n* = 26). See Tables 1, 2 for statistics. **(B,D)** No effect of Aβ-on sag **(B)** or sag ratio **(D)** was detected in wt littermates of GluN2B^+/−^ mice (*N* = 6, *n* = 29; *N* = 6, *n* = 31), although Aβ-caused an increase in sag ratio **(D)**, but not in sag **(B)** in GluN2B^+/−^ hippocampi (*N* = 6, *n* = 31) compared to GluN2B^+/−^ controls (*N* = 5, *n* = 28). See Tables 1, 2 for statistics. **(E,F)** Representative examples of sag in control peptide and Aβ-treated GluN2A^+/−^ transgenics and their wt littermates **(E)** and in control peptide and Aβ-treated GluN2B^+/−^ transgenics and their wt littermates **(F)**. The circles on the error bars show the distribution of individual responses in each condition that contributed the mean effect represented by the bar.

We detected an increased negativity of sag, but an unchanged sag ratio was observed in GluN2A wt littermates that were treated with Aβ compared to control peptide–treatment (Figures 3A,C; Table 1A). Sag and sag ratio were equivalent in control peptide–treated wt and control peptide–treated GluN2A^+/−^ (Figures 3A,C) and in Aβ-treated GluN2A^+/−^ compared to control peptide–treated transgenics (Figures 3A,C; Tables 1A, B). Thus, the only notable sag change we detected was in Aβ-treated wt littermates compared to control wt. In other words, GluN2A transgenics had altered sag but this was not further affected by Aβ.

In the GluN2B^+/−^ mice, sag was unchanged in Aβ-treated GluN2B^+/−^ hippocampi compared to hippocampi from control peptide–treatment GluN2B^+/−^ transgenics (Figure 3B; Table 2A). Sag ratio was significantly increased by Aβ-treatment, however (Figure 3D; Table 2A), suggesting that in Aβ-treated GluN2B^+/−^ transgenics, HCN channels may require a more negative membrane potential in order for them to become activated (see Figures 3E,F for representative examples).

### 3.4 Action potential properties were changed by Aβ-treatment in GluN2A^+/−^, but not GluN2B^+/−^ mice

**Figure 4:**
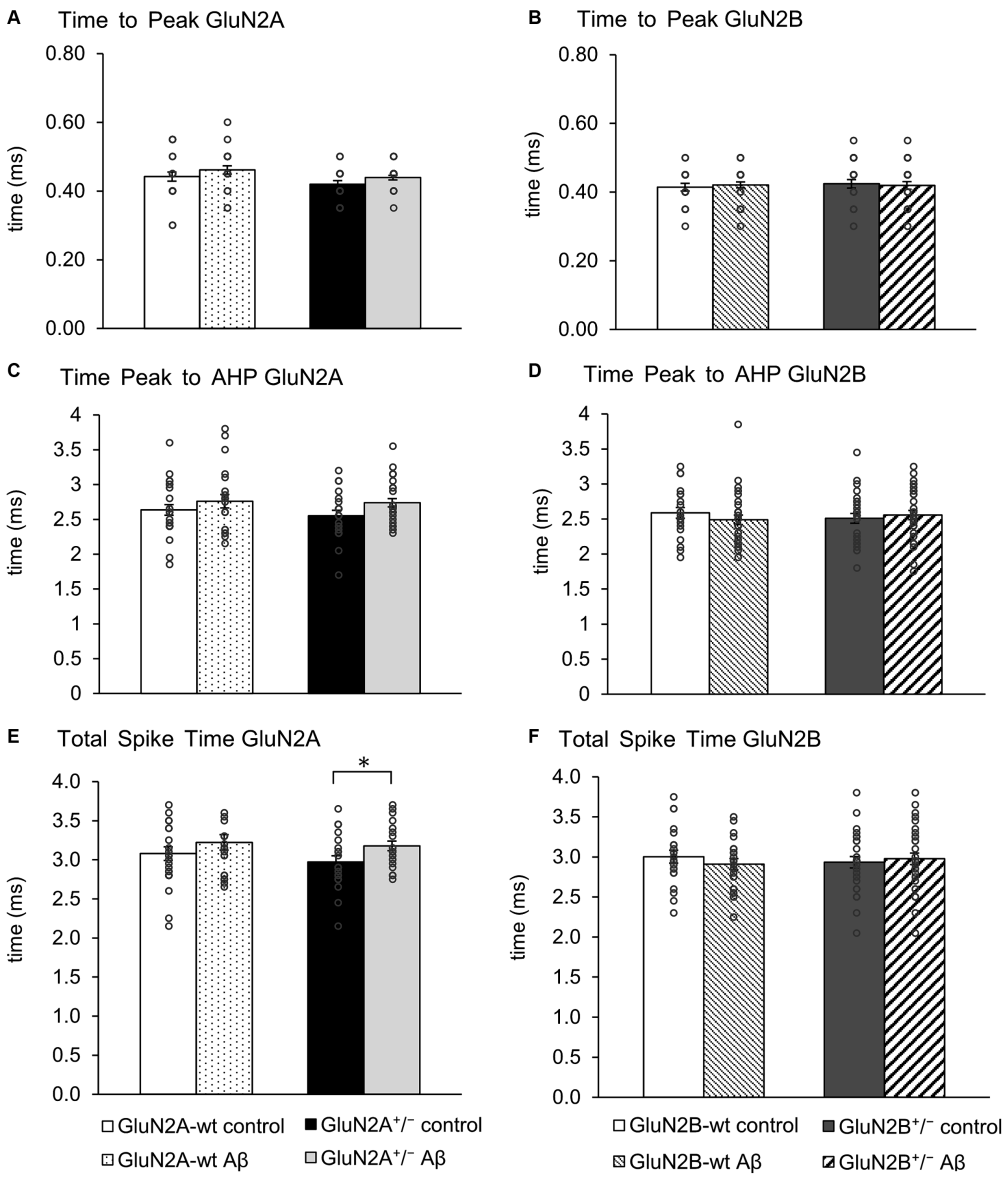
GluN2A^+/−^ but not GluN2B^+/−^ hippocampi showed sensitivity of spike time but not time to peak and AHP time, following Aβ-treatment. **(A,B)** Time to peak was unaffected by oligomeric Aβ (1–42)-treatment of GluN2A^+/−^ **(A)** and GluN2B^+/−^ mice **(B)** or their wt littermates **(A,B)** compared to control peptide-treated mice. See Table 3 for statistics. **(C,D)** The time from the peak of the action potential to the afterhyperpolarization (AHP) was not significantly increased by Aβ-treatment of GluN2A^+/−^ mice (*N* = 5, *n* = 25) **(C)** compared to control peptide-treated transgenic mice (*N* = 5, *n* = 20). Wildtype littermates were unaffected (*N* = 5, *n* = 25; *N* = 5, *n* = 22) **(C)**. No significant changes in the time from the peak of the action potential to the AHP were detected in GluN2B^+/−^ mice or their wt littermates after Aβ-treatment **(D)**. See Table 3 for statistics. **(E,F)** The total spike time was increased by Aβ-treatment of GluN2A^+/−^ mice **(E)** compared to control peptide-treated ko mice. Wildtype littermates were unaffected **(E)**. No significant changes in total spike time occurred in GluN2B^+/−^ mice or their wt littermates after Aβ-treatment **(F)**. See Table 3 for statistics. The circles on the error bars show the distribution of individual responses in each condition that contributed the mean effect represented by the bar.

Action potential properties (see Figures 2G,H for representative examples) such as time to peak (Figures 4A,B), time from peak to afterhyperpolarization (AHP) (Figures 4C,D), and total spike time (Figures 4E,F), were unaltered in wt littermates of GluN2A^+/−^ and GluN2B^+/−^ mice after Aβ-treatment, compared to responses evoked after treatment of wt with control peptide (Tables 3A, B). Time to peak was also unaffected by Aβ-treatment of either GluN2A^+/−^ (Figure 4A) or GluN2B^+/−^ transgenic mice (Figure 4B; Tables 3A, B) compared to control transgenic responses. The time of the peak to AHP was unchanged in Aβ-treated GluN2A^+/−^ compared to control peptide-treated GluN2A^+/−^ hippocampi (Figure 4C; Table 3A), and no Aβ-mediated effect was evident in Aβ-treated GluN2B^+/−^, compared to control peptide-treated GluN2B^+/−^ hippocampi (Figure 4D; Table 3B). Total spike time was significantly increased in Aβ-treated GluN2A^+/−^ compared to control peptide-treated GluN2A^+/−^ hippocampi (Figure 4E; Table 3A), but effects were absent in GluN2B^+/−^ compared to control peptide-treated GluN2B^+/−^ hippocampi (Figure 4F; Table 3B). Thus, only GluN2A^+/−^ hippocampi showed a sensitivity of the peak to AHP and the total spike time to oligomeric Aβ (1–42)-treatment.

**Table 3:** Action potential properties in GluN2A^+/−^ and GluN2B^+/−^ mice and their wt littermates following treatment with Aβ or control peptide.

A						
	GluN2A-wt control	GluN2A-wt Aβ	*T*-Test	GluN2A^+/−^ control	GluN2A^+/−^ Aβ	*T*-test
AP threshold (mV)	−36.20 ± 0.88	−34.90 ± 1.11	*p* = 0.37	−35.92 ± 0.96	−36.33 ± 0.86	*p* = 0.76
Spike amplitude (mV)	92.52 ± 1.26	90.52 ± 1.88	*p* = 0.38	93.12 ± 1.10	91.61 ± 1.16	*p* = 0.37
Time to peak (ms)	0.44 ± 0.01	0.46 ± 0.01	*p* = 0.298	0.42 ± 0.01	0.44 ± 0.01	*p* = 0.12
Time peak to AHP (ms)	2.64 ± 0.08	2.76 ± 0.10	*p* = 0.32	2.55 ± 0.08	2.74 ± 0.06	*p* = 0.07
Total spike time (ms)	3.08 ± 0.09	3.22 ± 0.10	*p* = 0.28	2.97 ± 0.08	3.18 ± 0.06	***p* = 0.046**
Ascending slope (mV/ms)	215.89 ± 9.4	200.5 ± 8.17	*p* = 0.24	224.87 ± 6.87	210.37 ± 4.74	*p* = 0.08
Descending slope (mV/ms)	39.27 ± 1.62	36.94 ± 1.56	*p* = 0.32	40.30 ± 1.34	36.57 ± 0.75	***p* = 0.015**
Half-width (ms)	0.93 ± 0.02	0.99 ± 0.02	*p* = 0.06	0.92 ± 0.02	0.98 ± 0.02	*p* = 0.052
20%-width (ms)	1.40 ± 0.04	1.49 ± 0.03	*p* = 0.054	1.38 ± 0.03	1.48 ± 0.03	***p* = 0.026**
AP peak (mV)	56.32 ± 0.71	55.63 ± 0.99	*p* = 0.57	57.20 ± 0.69	55.29 ± 0.53	***p* = 0.035**

B						
	GluN2B-wt control	GluN2B-wt Aβ	*T*-test	GluN2B^+/−^ control	GluN2B^+/−^ Aβ	*T*-test
AP threshold (mV)	−36.73 ± 1.28	−37.74 ± 0.87	*p* = 0.51	−36.66 ± 0.84	−38.18 ± 0.87	*p* = 0.23
Spike amplitude (mV)	95.87 ± 1.82	94.52 ± 1.24	*p* = 0.53	94.28 ± 1.46	96.88 ± 1.26	*p* = 0.19
Time to peak (ms)	0.41 ± 0.01	0.42 ± 0.01	*p* = 0.64	0.42 ± 0.01	0.42 ± 0.01	*p* = 0.78
Time peak to AHP (ms)	2.59 ± 0.07	2.49 ± 0.07	*p* = 0.35	2.51 ± 0.07	2.56 ± 0.07	*p* = 0.63
Total spike time (ms)	3.00 ± 0.08	2.91 ± 0.07	*p* = 0.399	2.93 ± 0.07	2.98 ± 0.07	*p* = 0.68
Ascending slope (mV/ms)	236.92 ± 10.84	229.35 ± 7.83	*p* = 0.57	229.24 ± 9.55	236.99 ± 8.03	*p* = 0.54
Descending slope (mV/ms)	41.42 ± 1.50	42.06 ± 1.26	*p* = 0.75	41.66 ± 1.38	41.75 ± 1.21	*p* = 0.96
Half-width (ms)	0.91 ± 0.03	0.88 ± 0.01	*p* = 0.42	0.92 ± 0.02	0.93 ± 0.03	*p* = 0.69
20%-width (ms)	1.35 ± 0.04	1.32 ± 0.02	*p* = 0.48	1.37 ± 0.03	1.39 ± 0.04	*p* = 0.68
AP peak (mV)	59.14 ± 0.92	56.78 ± 0.70	***p* = 0.048**	57.62 ± 0.83	58.70 ± 0.70	*p* = 0.33

(A) The table shows action potential properties of GluN2A^+/−^ mice and their wt littermates, after control peptide treatment or treatment with Aβ. AP, Action potential. Significant effects are highlighted in bold. (B) The table shows action potential properties of GluN2B^+/−^ mice and their wt littermates, after control peptide treatment or treatment with Aβ. AP, Action potential. Significant effects are highlighted in bold.

**Figure 5:**
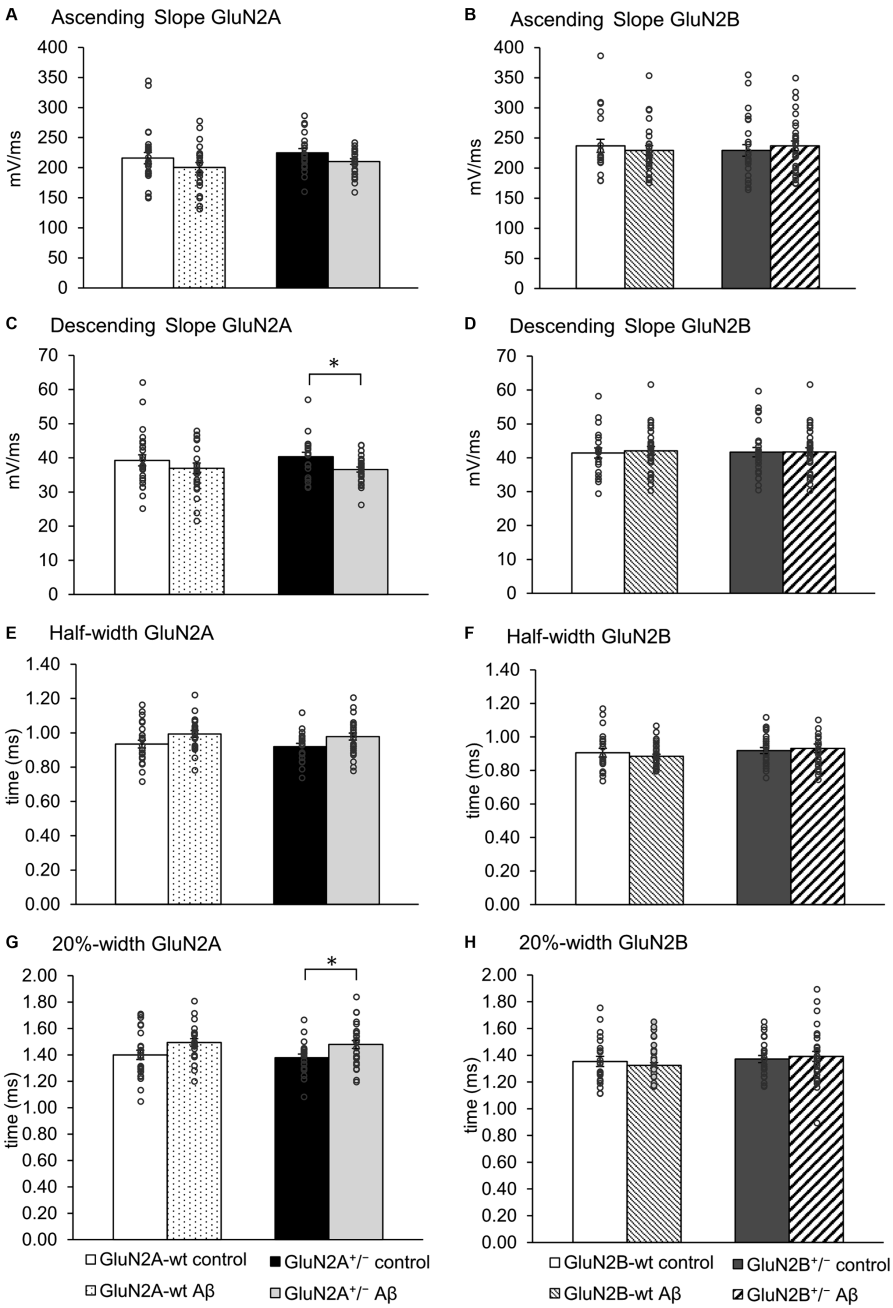
The action potential profile was altered in GluN2A^+/−^, but not GluN2B^+/−^ hippocampi following Aβ-treatment. **(A,B)** The ascending slope of the action potential (AP) was unaffected by Aβ-treatment in any of the groups. See Table 3 for statistics. **(C,D)** Aβ-treatment decreased the descending AP slope of GluN2A^+/−^ transgenics (*N* = 5, *n* = 25), compared to control GluN2A^+/−^ hippocampi (*N* = 5, *n* = 20) **(C)**. The descending slope **(D)** of the AP was not altered following Aβ-treatment of GluN2B^+/−^ transgenics, or their wt littermates compared to treatment with control peptide. **(E,F)** Aβ-treatment had no effect on the half-width of the AP in GluN2A^+/−^ **(E)** and GluN2B^+/−^ transgenics **(F)**, or their wt littermates compared to treatment with control peptide. See Table 3 for statistics. **(G,H)** Following Aβ-treatment, the 20% width **(G)** of the AP was increased in GluN2A^+/−^ mice compared to control peptide-treated transgenics. GluN2A wt littermates exhibited an unchanged 20%-width **(G)** after Aβ-treatment compared to control wt. Aβ-treatment had no effect on the 20% width of the AP in GluN2B^+/−^ transgenics, or their wt littermates compared to treatment with control peptide **(H)**. See Table 3 for statistics. The circles on the error bars show the distribution of individual responses in each condition that contributed the mean effect represented by the bar.

When we assessed the ascending and descending slope of the action potential, we found no changes following Aβ-treatment of wt littermates compared to control peptide treatment of wt (Figures 5A–D; Tables 3A, B). No significant changes in the ascending slope were detected following Aβ-treatment of GluN2A^+/−^ mice, compared to control peptide-treatment of GluN2A^+/−^ mice (Figure 5A; Table 3A). The descending slope was significantly slower, however (Figure 5A). No differences in ascending (Figure 5B), or descending, slope (Figure 5D) were detected in GluN2B^+/−^ hippocampi following Aβ–treatment (Table 3B).

The half width (Figure 5E) and 20%-width (Figure 5G) of the action potential was unchanged following Aβ-treatment of wt littermates of the GluN2A^+/−^ mice (Table 3A). Effects were significant (20%-width) following Aβ-treatment of GluN2A^+/−^ mice compared to control peptide effects (Figures 5E,G; Table 3A). Thus, Aβ-treatment altered the width of the action potential in GluN2A^+/−^ mice.

In GluN2B^+/−^ mice or their wt littermates, Aβ-treatment had no effect on the width of the action potential (Figures 5F,H; Table 3B). No differences in Aβ-treatment effects were evident when the two wt cohorts were compared.

Taken together, the slowing of the action potential may serve to explain why the time to AHP and the total spike time was increased in Aβ-treated GluN2A^+/−^ mice (Figures 4C,E). Aβ-treatment had no effect whatsoever on action potential properties in GluN2B^+/−^ and their wt littermates.

### 3.5 Action potential firing frequency was not altered in GluN2A^+/−^ and GluN2B^+/−^ mice compared to wildtype littermates. Aβ-treatment had no effect

**Figure 6:**
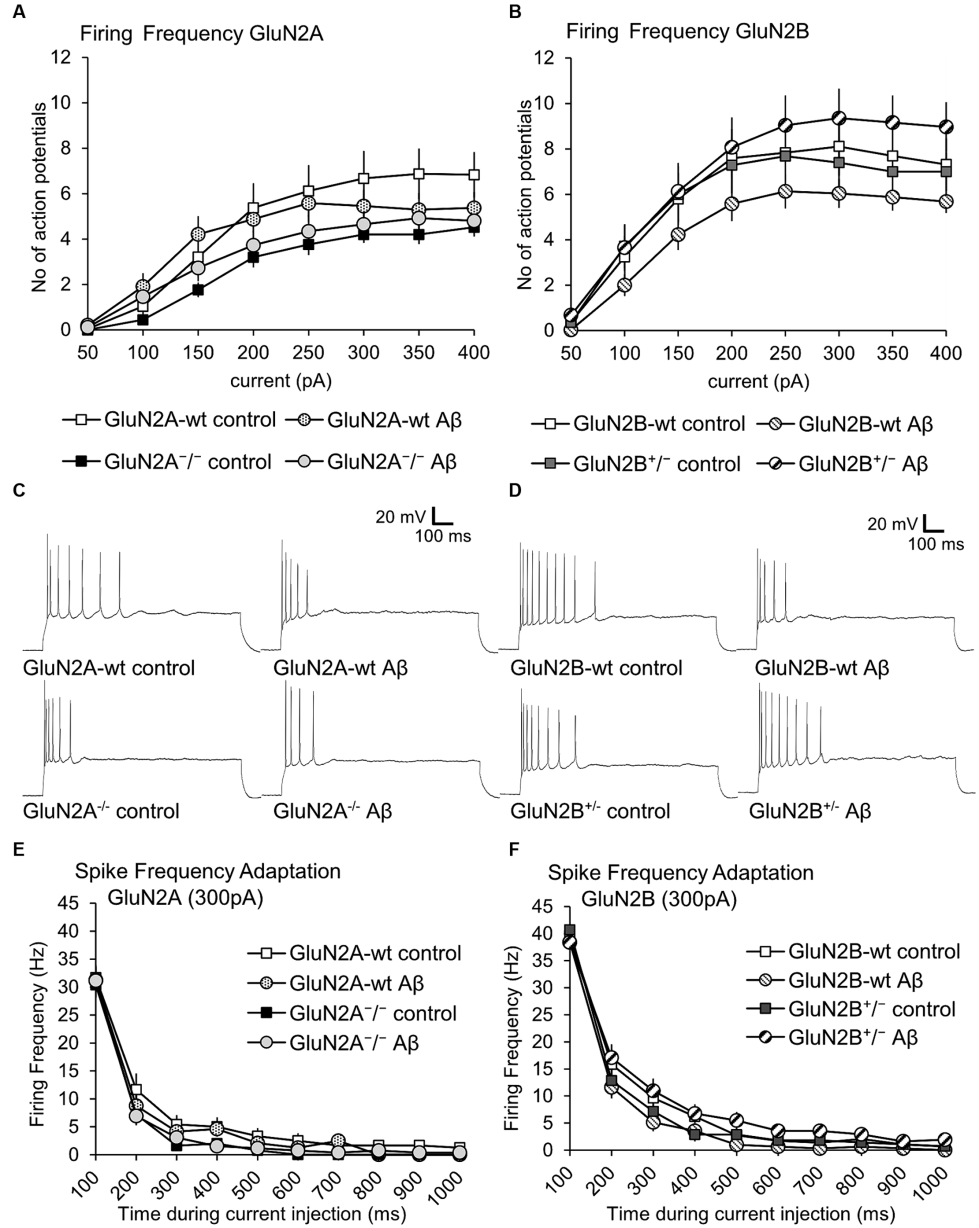
Firing frequency was not altered by Aβ-treatment of GluN2A^+/−^ mice, GluN2B^+/−^ mice and their wt littermates. **(A,B)** Firing frequency (FF) was not altered in control peptide-treated GluN2A^+/−^ (*N* = 5, *n* = 25) or GluN2B^+/−^ (*N* = 5, *n* = 28) transgenics compared to their wildtype (wt) littermates (*N* = 5, *n* = 24; *N* = 6, *n* = 29). See Tables 1, 2 for statistics. **(C)** Analog examples of action potential trains by a current intensity of 300pA in control peptide–treated wildtype (wt) littermates of GluN2A^+/−^ mice (top left) and Aβ-treated wt littermates (top right), as well as control peptide–treated GluN2A^+/−^ mice (bottom left), and Aβ-treated GluN2A^+/−^mice (bottom right). **(D)** Analog examples of action potential trains by a current intensity of 300pA in control peptide–treated wildtype (wt) littermates of GluN2B^+/−^ mice (top left) and Aβ-treated wt littermates (top right), as well as control peptide–treated GluN2B^+/−^ mice (bottom left), and Aβ-treated GluN2B^+/−^ mice (bottom right). **(E,F)** Spike frequency adaptation at 300 pA was not altered in control peptide-treated GluN2A^+/−^ (*N* = 5, *n* = 25) or GluN2B^+/−^ (*N* = 5, *n* = 28) transgenics compared to their wildtype (wt) littermates (*N* = 5, *n* = 24; *N* = 6, *n* = 29).

When we compared action potential firing frequency and spike frequency adaptation in control peptide-treated GluN2A^+/−^ mice and their wt littermates, we detected no significant differences (Figures 6A,E; Table 1B). Treatment with Aβ failed to alter firing frequency, or spike frequency adaptation in either wt or GluN2A^+/−^ hippocampi (Figures 6A,E; Tables 1A, 4).

**Table 4:** Summary of effects of oligomeric Aβ (1–42) or control peptide-treatment on passive and active neuronal membrane properties of hippocampal pyramidal cells.

	Aβ versus control peptide				Control versus control		Aβ versus Aβ	
	GluN2A wt × wt	GluN2A ko × ko	GluN2B wt × wt	GluN2B ko × ko	GluN2A wt × ko	GluN2B wt × ko	GluN2A wt × ko	GluN2B wt × ko
Resting potential	–	↑	–	–	–	–	–	–
Input resistance	↑	–	–	–	–	–	–	–
Excitatory threshold	–	–	–	–	–	–	–	–
Sag	↑	–	–	–	–	–	–	–
Sag ratio	–	–	–	↑	–	–	–	–
Time to peak	–	–	–	–	–	–	–	–
Peak to AHP	–	–	–	–	–	–	–	–
Total spike time	–	↑	–	–	–	–	–	–
Ascending slope	–	–	–	–	–	–	–	–
Descending slope	–	↓	–	–	–	–	–	–
Half-width	–	–	–	–	–	–	–	–
20%-width	–	↑	–	–	–	–	–	–
Firing frequency	–	–	–	–	–	–	–	–

In all cases, the upward-pointing arrows indicate an increase, and the downward-pointing arrows indicate a decrease in values in the control condition. A dash signifies no effect. AHP, afterhyperpolarization.

No differences in firing frequency (ANOVA *F*(3,115) = 1.2631, *p* = 0.29), or spike frequency adaptation were detected between GluN2B^+/−^ mice and their wt littermates after control peptide-treatment (Figures 6B,F; Table 2B). Although a tendency towards increased firing frequency and higher currents was evident after Aβ-treatment of GluN2B^+/−^ mice, this was not significant compared to control peptide-treated transgenics (Figure 6B; Tables 2A, 4).

Thus, Aβ-treatment had no effect on firing frequency in GluN2A^+/−^ or GluN2B^+/−^ mice and their wildtype littermates (see Figures 6C,D for representative examples).

### 3.6 LTP duration was differentially curtailed in GluN2A^+/−^ or GluN2B^+/−^ hippocampi. LTP was impaired by Aβ-treatment of wt littermates. LTP was not further altered by Aβ-treatment of GluN2A^+/−^ or GluN2B^+/−^ mice

**Figure 7:**
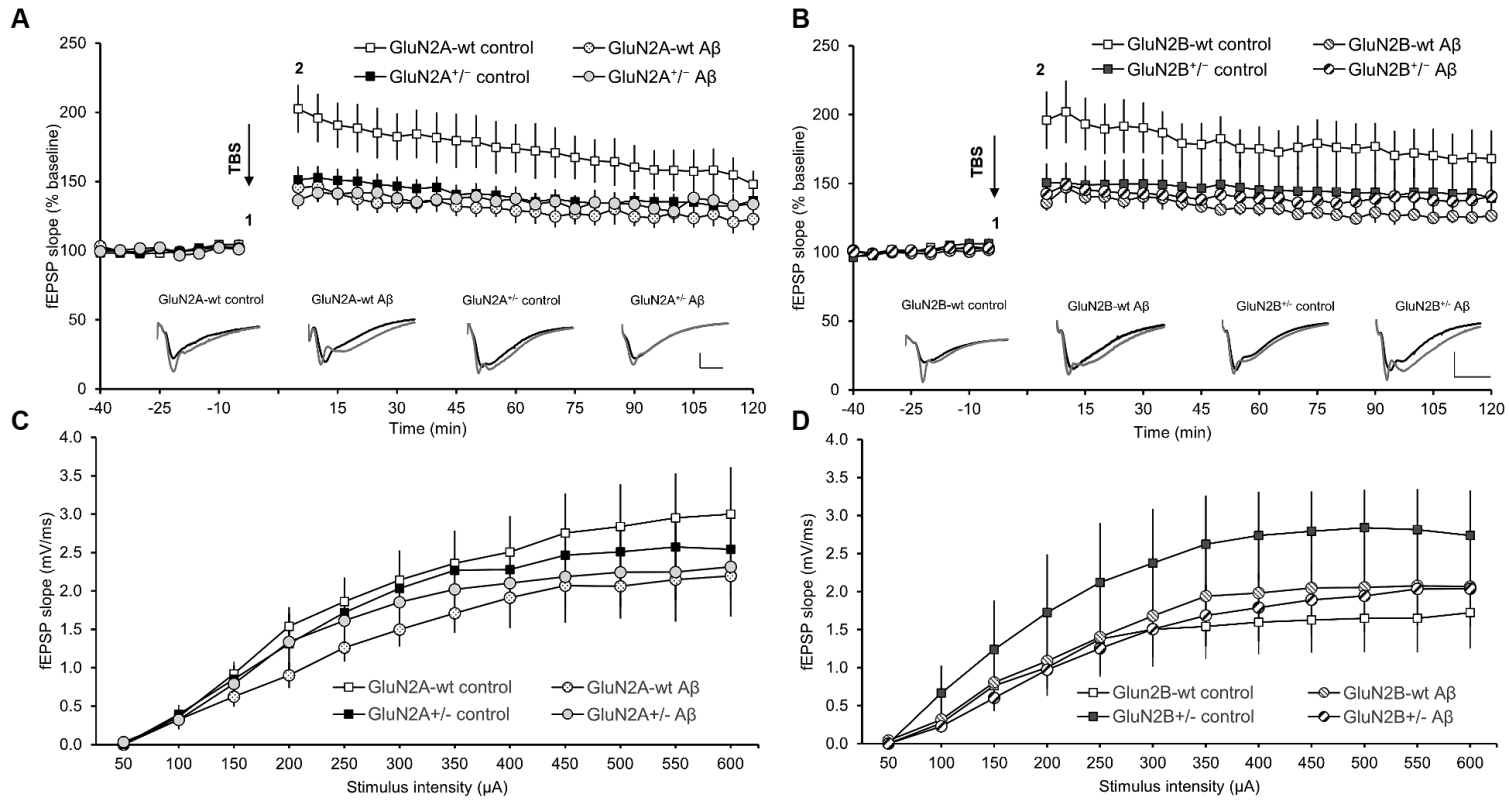
Deficits in LTP that were elicited in wild-type hippocampi by oligomeric Aβ (1–42) were absent in GluN2A^−/+^ and GluN2B^−/+^ hippocampi. The stimulus–response relationship is unaffected transgenic alteration or Aβ (1–42)-treatment. **(A,B)** Four to 5 weeks after Aβ–treatment, the early phase of hippocampal LTP was significantly impaired compared to control peptide-treated wt littermates of GluN2A^+/−^ **(A)** or GluN2B^+/−^-transgenic mice **(B)**. By contrast, LTP in GluN2A^+/−^ **(A)** or GluN2B^+/−^ transgenic hippocampi **(B)** was unaffected by Aβ–treatment, with responses being equivalent in Aβ–treated tg compared to control peptide-treated tg or Aβ–treated wt slices. Insets in A and B show analog examples of potentials evoked 5 min prior to (1) and 5 min after (2) theta burst stimulation (TBS). Scale bars represent 1 mV in the vertical, and 5 ms in the horizontal, axis. The stimulus–response relationship (obtained in steps ranging from 50 through 600 μA) was not significantly different in GluN2A^+/−^ **(C)** or GluN2B^+/−^ transgenic hippocampi **(D)**, compared to their wildtype littermates, after treatment with oligomeric Aβ (1–42) or control peptide.

LTP, induced by theta-burst stimulation (TBS), was significantly impaired in the hippocampi of control-peptide treated GluN2A^+/−^transgenic mice (*N* = 7, *n* = 9) compared to their wt littermates (control-peptide treated) (*N* = 6, *n* = 9) (Figure 7A). Impairments were evident throughout the entire monitoring period and were still evident 30 min (ANOVA *F* (1, 15) = 6.67, *p* = 0.02), 60 min (ANOVA *F* (1, 15) = 5.88, *p* = 0.03) and 120 min post-TBS (ANOVA *F* (1, 15) = 5.09, *p* = 0.04) (Figure 7A).

Treatment of GluN2A^+/−^ transgenic mice with Aβ (1–42) (*N* = 6, *n* = 7) resulted in LTP that was not statistically different from LTP elicited in control peptide-treated transgenics (*N* = 7, *n* = 9) (Figure 7A) (ANOVA 30 min post-TBS: *F* (1, 14) = 0.80, *p* = 0.39). Thus, the impairment of LTP that was evident in wt littermates, was not present in GluN2A^+/−^hippocampi. Rather the reduced LTP that occurred in control peptide-treated GluN2A^+/−^ transgenics was not further exacerbated by Aβ (1–42)-treatment.

In GluN2B^+/−^ transgenics (*N* = 6, *n* = 6), the early phase of LTP, induced by TBS, was significantly impaired in the hippocampi of GluN2B^+/−^ transgenic mice that had been treated with control peptide (*N* = 7, *n* = 9), compared to their wt littermates (*N* = 7, *n* = 8) (Figure 7B). Impairments were sustained until 15 min post-TBS (*p* = 0.04). Thereafter, responses exhibited increased variability. The entire monitoring period of LTP was significantly impaired in wt littermates following Aβ (1–42)-treatment (*N* = 7, *n* = 8) compared to wt that had been treated with control peptide (*N* = 7, *n* = 8), with effects being immediately apparent after TBS, and sustained at 30 min (ANOVA *F* (1, 13) = 8.199, *p* = 0.013), 60 min (ANOVA *F* (1, 13) = 9.11, *p* = 0.0098) and 120 min post-TBS (ANOVA *F* (1, 13) = 8.65, *p* = 0.011) (Figure 7B). By contrast, treatment of GluN2B^+/−^ with Aβ (*N* = 7, *n* = 8) resulted in LTP that was not significantly different from LTP evoked in control peptide-treated GluN2B^+/−^hippocampi (*N* = 6, *n* = 6) (ANOVA 30 min post-TBS: *F* (1, 12) = 0.24, *p* = 0.63).

No significant changes were detected in the stimulus–response relationship when treatment conditions were compared in GluN2A^+/−^mice (*N* = 6, *n* = 8) and their wt littermates) (*N* = 6, *n* = 9) (Figure 7C), or in GluN2B^+/−^ transgenics (*N* = 6, *n* = 6) and their wt littermates (*N* = 7, *n* = 8) (Figure 7D). Thus, treatment with Aβ did not alter the synaptic response to afferent stimulation.

Taken together, these results indicate that whereas GluN2A is required for prolonged LTP induced by TBS, under these afferent stimulation conditions GluN2B supported only the early phase of LTP. Treatment with oligomeric Aβ (1–42) significantly impaired LTP in wt mice. However, transgenic knockdown of GluN2A, or GluN2B, did not exacerbate the debilitating effects of Aβ on LTP in aging mice.

## 4 Discussion

In this study, we report that in 8–15 month old animals, neuronal properties were largely equivalent in the hippocampi of GluN2A^+/−^ and GluN2B^+/−^ transgenic mice compared to their wt littermates (Table 4). A limited range of changes in properties of CA1 pyramidal cells were detected 4–5 weeks following intracerebral oligomeric Aβ (1–42)-treatment of wildtype animals, comprising, for example, a higher input resistance and a more negative sag, in wt littermates of GluN2A^+/−^ mice (Table 4). Aβ-treatment elicited a limited amount of changes in neuronal properties of the transgenic animals, whereby GluN2A^+/−^ mice were more affected than GluN2B^+/−^ mice (Table 4). LTP was impaired in both GluN2A^+/−^ and GluN2B^+/−^ hippocampi compared to their wt littermates. Furthermore, intracerebral treatment with oligomeric Aβ (1–42) resulted in an impairment of LTP in wt mice. Strikingly, the profile of LTP was unchanged in Aβ-treated GluN2A^+/−^ or GluN2B^+/−^ hippocampi compared to control peptide-treated transgenic hippocampi, meaning that the already deficient LTP (compared to wt) was not impaired further by Aβ-treatment. Taken together, these data indicate that in the aging brain, GluN2A-containing NMDAR played an important role in the homeostasis of neuronal excitability. Furthermore, neuronal function was only mildly affected by Aβ-treatment of aging wt or GluN2 deficient mice, and knockdown of GluN2A or GluN2B did not worsen the debilitating effects of oligomeric Aβ (1–42) on hippocampal LTP. This suggests that in the aging hippocampus, NMDAR were not instrumental in propagating the pathophysiological effects of oligomeric Aβ (1–42) on hippocampal function.

By and large, we detected no marked effects of Aβ-treatment on neuronal properties. We saw for example, a greater positivity of the resting membrane potential in GluN2A^+/−^ hippocampi compared to control GluN2A^+/−^ hippocampi, but no significant difference in the membrane potential in GluN2B^+/−^ compared to control GluN2B^+/−^ hippocampi. The former difference derived more from differences in control peptide effects in wt and transgenics than from direct effects of Aβ, however. Sag (I_h_) ratio was increased in GluN2B^+/−^ following Aβ-treatment but unaffected in GluN2A^+/−^ hippocampi following treatment. The I_h_ stabilises the resting membrane potential, regulates the afterhyperpolarization and influences firing frequency (McCormick and Pape, 1990). Neuronal oscillations are supported by the I_h_ (McCormick and Pape, 1990; Wahl-Schott and Biel, 2009) and we have reported in the past that hippocampal neuronal oscillations are undermined by oligomeric Aβ (1–42) (Kalweit et al., 2015). Sag is enabled by HCN channels (Robinson and Siegelbaum, 2003). HCN channels modulate glutamate release in the hippocampus and thus, influence NMDAR currents (Neitz et al., 2014). It has been proposed that these channels support hippocampal plasticity processes (Honnuraiah and Narayanan, 2013). NMDAR-dependent spontaneous slow excitatory dendritic potentials are regulated by HCN channels and are mediated by GluN2B-containing NMDARS (Ashhad and Narayanan, 2016). Others have reported a more positive resting membrane potential and enhanced I_h_ current in the hippocampus after intracerebral Aβ-treatment (Eslamizade et al., 2015). Our finding that sag ratio was more positive after Aβ-treatment of GluN2B^+/−^ mice, suggesting that GluN2B contributed to these effects.

A role for GluN2B has been described in Aβ-mediated effects in the hippocampus: The reduction in network activity and LTP that occurs following topical application of Aβ to hippocampal slices or neuronal cultures, or intracerebral treatment of rats is prevented by antagonists of GluN2B (Hu et al., 2009; Rönicke et al., 2011). It has also been reported that the enhancement of NMDAR currents and intracellular calcium levels that occur following application of Aβ are mediated by GluN2B-containing NMDAR (Li et al., 2011; Ferreira et al., 2012). Furthermore, Aβ (1–40) triggers an increase in the expression of GluN2B in hippocampal neuronal cultures (Chang et al., 2016) and the GluN2A:GluN2B ratio decreases after Aβ (1–42) application (Huang et al., 2017). Interestingly, inhibition of GluN2B-containing NMDAR prevents Aβ-mediated impairments of LTP (Rönicke et al., 2011; Huang et al., 2017). The frequency-dependency of LTP was not assessed in these studies, but our findings suggest that less potent forms of LTP that do not critically require activation of GluN2B are not affected by Aβ (1–42). This is all the more interesting given the advanced age of the mice in our study: all of the studies mentioned above used young adult animals and treatment regimes of maximally 15 days before hippocampal scrutiny.

We previously reported that the frequency of the afferent input, and impulse number it delivers, determines the recruitment of GluN2A or GluN2B subunit-containing NMDAR to LTP in the CA1 region (Ballesteros et al., 2016). Weak afferent stimulation recruits a GluN2A-dependent form of LTP that is small in magnitude and short (>4 h) in duration. By contrast, strong afferent stimulation recruited LTP that required GluN2B-containing NMDAR that was much larger in magnitude and lasted over 24 h (Ballesteros et al., 2016). The form of LTP examined in the present study had both a GluN2A and a GluN2B-dependent component, as indicated by the reduction in LTP magnitude in GluN2A and GluN2B transgenic hippocampi compared to wt littermates. Effects were more potent in GluN2A transgenics, although the increased variability in responses in the later phase of LTP in GluN2B transgenics may have masked deficits in LTP compared to controls. Although we cannot entirely rule out the possibility that LTP was successfully induced, albeit less potently, in the GluN2A^+/−^ or the GluN2B^+/−^ mice by non-ionotropic mechanisms, this seems unlikely. Theta-burst stimulation induces a decremental form of LTP in the mouse hippocampal slice preparation that is distinct from more robust forms of LTP induced by high frequency afferent stimulation (Novkovic et al., 2015). The recruitment of, for example, voltage-dependent calcium channels into hippocampal LTP requires very fast high frequency stimulation (Grover and Teyler, 1990; Manahan-Vaughan et al., 1998) and forms of synaptic potentiation that can be induced by activation of metabotropic glutamate (mGlu), or catecholaminergic, receptors are temporally slow to become manifest (Manahan-Vaughan and Reymann, 1995; Tse et al., 2023) and do not fit the temporal dynamics of the LTP profiles induced in our study. We did not see a complete abolishment of LTP in the GluN2A^+/−^, or the GluN2B^+/−^ hippocampi presumably because the remaining subunits permitted a weaker form of LTP to occur. Evidence for this has been offered by pharmacological studies that showed that LTP, short-term potentiation and forms of synaptic depression can be induced with the same afferent stimulation frequency combined with a graded degree of activation of NMDAR (Cummings et al., 1996).

Forms of LTP that are intrinsically linked to learning are enabled by weak afferent activity in the hippocampus (Kemp and Manahan-Vaughan, 2004, 2008; Hagena and Manahan-Vaughan, 2012; Hoang et al., 2021). By contrast, very strong afferent stimulation induces robust LTP that is associated with reduced learning flexibility, reduced reversal learning and an absence of differentiated neuronal encoding in the hippocampus of rats (Barnes, 1979; Barnes et al., 1994; Hoang et al., 2021). This raises the question as to the functional requirement of GluN2B-dependent LTP in adulthood. It has been reported that the expression of GluN2-subunits declines after early postnatal development (Carmignoto and Vicini, 1992), but in adult C57Bl/6 mice, we did not observe an appreciable decline in receptor expression (Beckmann et al., 2020). This would suggest that both GluN2A and GluN2B-containing NMDAR contribute to LTP in adulthood. The kind of LTP (magnitude, persistency) may be determined by the kind of information that is encoded, however.

We previously reported that intracerebral treatment with oligomeric Aβ (1–42) 1 week prior to assessing LTP in 4–10 month old wildtype mice impairs the early phase of LTP (Südkamp et al., 2021). Animals in the present study were 8–15 months old at the time of treatment. Here, wildtypes showed a significant LTP impairment that extended to the later phases of plasticity. One possibility is that the increased age of the wildtypes may have caused a greater vulnerability to the debilitating effects of intracerebral Aβ-treatment. Age-dependent changes in hippocampal function have been reported (Barnes et al., 1994; Wilson et al., 2004; Twarkowski et al., 2016) that could underlie these effects. Another reason for the greater vulnerability of wildtype hippocampi in this study might be the enhanced time-period of exposure to Aβ-treatment. One cannot exclude, however, that although the background strain was identical for the wt mice in this and the abovementioned study (C57BL/6)(Sakimura et al., 1995; von Engelhardt et al., 2008; Dvoriantchikova et al., 2012), substrain-dependent differences influenced the outcome of Aβ-sensitivity. Genetic drift related to separation of breeding pools is likely to have an impact on the precise genomic identity of wildtype littermates derived from the C57BL/6 strain (Manahan-Vaughan, 2018), that could have influenced the sensitivity of the wildtypes to Aβ. For this reason, we included separate wt cohorts for both transgenic lines, whereby only wildtype littermates of either the GluN2A^+/−^ or the GluN2B^+/−^ were used.

The relatively mild effects of oligomeric Aβ (1–42) on neuronal responses, as detected by patch clamp in our study, corresponds to reports that the peptide predominantly affects synaptic transmission and thus, dendritic responses (Shankar et al., 2008). It has been reported that application of oligomeric Aβ (1–42) onto hippocampal slices from young (P26-32) rats results in an acute increase in surface expression of the GluA1 subunit of α-amino-3-hydroxy-5-methyl-4-isoxazolepropionic acid receptors (AMPAR) (Whitcomb et al., 2015). By contrast, topical application of oligomeric Aβ to hippocampal cultures from P18 rats inhibits AMPAR trafficking (Rui et al., 2010) and reduces surface expression of AMPAR (Guntupalli et al., 2017). Reductions in GluA1 density in the hippocampus have also been reported 30 days after intracerebral inoculation of 18 month old mice with Aβ (1–42) in a dose of 20 μM (Yeung et al., 2020). Scrutiny of the stimulus–response relationship of wildtype 8–15 month old mice that had been treated intracerebrally 4–5 weeks previously with 10 μM oligomeric Aβ (1–42) did not reveal any significant effects. The absence of deficits in the stimulus–response relationship that would indicate that AMPAR density had declined in Aβ (1–42)-treated wildtypes, may reflect differences in the oligomer doses used these two studies, or may indicate that although AMPAR density might have declined in the Aβ (1–42)-treated mice, this did not have a functional impact on synaptic transmission.

The maintenance of later phases of LTP beginning at around 90 min post-induction, are supported by phospholipase -C coupled (group 1) receptors (Hagena and Manahan-Vaughan, 2022; Mukherjee and Manahan-Vaughan, 2023). Group 1 mGlu receptors can functionally interact with NMDAR and alter NMDAR currents (Rosenbrock et al., 2010). Correspondingly pharmacological antagonists of group 1 mGlu receptors can alter the induction profile of hippocampal LTP (Neyman and Manahan-Vaughan, 2008). Aberrant mGlu5 receptor signaling is triggered by interactions of the receptor with Aβ (1–42) (Haas and Strittmatter, 2016) and mGlu5 receptors contribute to impairments of hippocampal LTP in 8–11 week old rats that are caused by acute cerebral treatment with Aβ (1–42) (Wang et al., 2004; Hu et al., 2014). Others have reported that Aβ (1–42) can form a complex with GluN2B and mGlu1 receptors (Taniguchi et al., 2022) and that mGlu5 receptors can bind with prion protein that serves as a target for Aβ (1–42) (Hu et al., 2014). We did not see any exacerbation of LTP deficits in GluN2A^+/−^ or the GluN2B^+/−^ mice that were treated with Aβ (1–42). This raises the interesting question as to whether the knockdown of NMDAR subunits left fewer interaction partners for the putative creation of an Aβ-prion protein-mGlu5 complex (Hu et al., 2014), or an Aβ-GluN2B-mGlu1 complex (Taniguchi et al., 2022), that would otherwise serve to further disrupt LTP in the transgenics.

## 5 Conclusion

In conclusion, this study shows that knockdown of GluN2A or GluN2B did not elicit substantial changes in neuronal properties within the hippocampus of aging (8–15 month old) mice. Nonetheless, loss of GluN2A appeared to result in a greater degree of change in neuronal properties suggesting that this subunit is more relevant than GluN2B for neuronal homeostasis in the aging hippocampus. Intracerebral treatment with oligomeric Aβ (1–42) 4–5 weeks before testing resulted in some changes in neuronal properties, but these were mostly apparent in GluN2A transgenics and a comparison of Aβ-mediated effects in the GluN2 transgenics and their wt littermates showed that responses were equivalent, suggesting that the knockdown of the subunits only slightly increased the vulnerability of the hippocampus to oligomeric Aβ (1–42). A similar profile was apparent with regard to hippocampal LTP: knockdown of GluN2A or GluN2B significantly impaired LTP in wt littermates. Treatment with oligomeric Aβ (1–42) resulted in an impaired LTP in wt littermates that was equivalent in magnitude to LTP in GluN2 transgenics. This impaired LTP was not debilitated further by Aβ (1–42)-treatment. The findings of this study show that although the aging hippocampus was affected by the intracerebral presence of oligomeric Aβ (1–42), and although knockdown of GluN2A or GluN2B impaired LTP, changes in the composition of the NMDAR did not contribute appreciably to the effects on neuronal properties or LTP caused by oligomeric Aβ (1–42). In sum, a loss of GluN2 subunit content in the hippocampus did not increase the vulnerability of this structure to the debilitating effects of oligomeric Aβ (1–42).

## Data availability statement

The raw data supporting the conclusions of this article will be made available by the authors, without undue reservation.

## Ethics statement

The animal study was approved by Landesamt für Arbeitsschutz, Naturschutz, Umweltschutz und Verbraucherschutz, NRW, Germany. The study was conducted in accordance with the local legislation and institutional requirements.

## Author contributions

NS: Formal analysis, Investigation, Methodology, Writing – review & editing, Data curation. OS: Formal analysis, Investigation, Methodology, Writing – review & editing, Data curation. DM-V: Conceptualization, Formal analysis, Funding acquisition, Methodology, Resources, Supervision, Writing – original draft, Writing – review & editing.
